# Overview of Application of Nanomaterials in Medical Domain

**DOI:** 10.1155/2022/3507383

**Published:** 2022-05-04

**Authors:** Jiabo Lan

**Affiliations:** School of Pharmacy, University College London, London WC1E 6BT, UK

## Abstract

With the development of nanotechnology, the application of nanomaterials in the medical field has become a forefront hotspot in the field of scientific research in the 21st century. Compared with traditional drug carriers, drug carriers made of nanomaterials have advantages such as higher drug loading rate, better biocompatibility, and targeted transportation, which provide the possibility for the treatment of a variety of diseases. In this paper, the characteristics and advantages of nanomaterials as well as their applications in the medical field are reviewed and the research progress of nanomaterials is analyzed.

## 1. Introduction

Nanotechnology, as the key scientific research field invested in by various countries since the 21st century, covers the technological innovation of building materials, computer chips, health care, environmental protection, new energy development, electric power transportation, and so on. In the development of modern medicine, for traditional drugs, it is one-stop and difficult to control the releasing time and positions. However, nanodrugs can improve the biocompatibility and the targeting ability of drugs and can increase the bioavailability and reduce the toxic and side effect of drugs. For example, the present new antineoplaston drugs, antiviral drugs, and rheumatoid arthritis drugs have been introduced with nanomedicine delivery systems to some extent, to improve their effectiveness. At present, the academic circle is still developing and optimizing the selection of nanomedicine materials and the control methods of drug release. In this paper, the materials and application scope of nanomedicine are reviewed to provide cutting-edge research information.

## 2. Characteristics and Advantages of Nanomaterials

Nanomaterials mainly refer to some particles with certain physical or chemical properties or biological effects, whose external size or internal size or surface structure are within the nanoscale range (1 nm∼100 nm) [1]. Nanomaterials can be divided into organic and inorganic nanomaterials, of which organic nanomaterials include nanofibers, nanotubes, liposomes, and polymer nanoparticles and inorganic nanomaterials include elementary substances, alloys, silica, and quantum dots [2].

Nanomaterials can effectively transport and load drugs since they have the largest specific surface area among all the known materials at present. At the same time, nanomaterials have good biocompatibility and biodegradability, and they can accumulate in human organs with less side effects [3]. In addition, nanomaterials have the properties of slow release, which can reduce drug concentration and toxic side effects [4]. Compared to traditional drugs which are with defects such as being ubiquitous in poor stability, apt to deform and inactivate, short biological half-life, and low bio-availability, and unable to easily go through the physiological barrier, biological nanomaterials play a role that cannot be ignored in the field of biological medicine, such as in diagnosis, treatment, repair, or replacement of the damaged organization. For example, the nanoparticles with small size are easy to be swallowed up by cells; nanodrugs of large specific surface area and more functional groups or active centers can realize a large load of specific drugs; nanomaterials with the characteristics of porous, hollow, multilayer structures are easy for control and release of drugs, so as to change the half-life period of drugs in the body and prolong the action time of drugs.

With the deepening of research on nanomaterials, nanomaterials have been developed from being only the delivery carrier of drugs to a new type of materials which are with certain biological effects and can participate in the treatment of diseases [5]. With the continuous innovation of nanomaterials, the physicochemical properties and structural characteristics of nanodrugs are enriched and the multifunctional nanomaterials have great application potential in the field of biomedicine.

## 3. Application of Inorganic Nanomaterials in the Medical Field

### 3.1. Application of Elemental Nanomaterials in the Medical Field

As a new inorganic nanomaterial, selenium nanoparticles (SeNPs) were prepared after using chitosan ascorbic acid to restore sodium selenite, with oligosaccharide (COS) as the template. Se was physically adsorbed on the surface of chitosan oligosaccharide in an amorphous form by virtue of its characteristics of good water solubility and rapid degradation.

As drug-loaded chitosan nanoparticles are only slightly dissolved in dilute acid, when they degrade in the human body, the acidic substances will also accumulate and generate adverse effects in the human body. Therefore, how to improve the water solubility of chitosan and its degradability in organisms is a very important issue. Since SeNP adopted COS which is of good water solubility, high biological activity, and easy absorption for the human body, instead of chitosan which needs to be dissolved with acetic acid [6], residue solvent toxicity caused by acetic acid can be avoided. And, the prepared selenium nanoparticles are of uniform size, good dispersibility, and have good inhibition to cancer cells under high concentration. The inhibition of them is stronger, with higher concentration. According to the study of Tong Chunyi et al. [7], when HCC SMMC-7721 cells were treated with SeNP, cell growth was promoted at low concentration (below 5.68 × 10–5 M), while cell growth was inhibited at high concentration (above 6.77 × 10^−5^ M) and the inhibition rate increased with the increase in selenium content. When the concentration was 7.50 × 10^−4^ M, the inhibition rate reached about 70%.

In Ren's study [8] on rheumatoid arthritis (RS), through the treatment on rats of the RA model with SeNPs, it was found that SeNPs played their role of anti-inflammatory action by regulating the expression of glutathione peroxidase (GSH-Px), rabbit antirat cyclooxygenase-2 (COX-2), and tumor necrosis factor-*α* (TNF-*α*). The condition of rats with the RA model was better.

### 3.2. Application of Mesoporous Silica in the Medical Field

Mesoporous silica nanoparticles (MSN_S_), as a new type of inorganic nanomaterial, mainly taking advantage of their characteristics of large mesoporous surface and high specific surface area, interact with drugs and realize drug delivery through ionic bonding, hydrogen bonding, and electrostatic interaction [9]. At the same time, it is easy to regulate pore volume and aperture to load drug into the MSN mesoporous channels, so as to control the release process of drug [10].

However, due to the unmodified MSN material characteristics of easy aggregation, poor targeting, and poor dispersion in aqueous solution, the application of MSNs in tumor therapy has been limited to a certain extent. In order to improve this situation, Xiao et al. modified the hydrophobic alkyl chain (C18) on the surface of MSNs based on disulfide bond, used its hydrophobic effect to coat amphiphilic polypeptide (AP) containing RGD ligand on the surface of MSNs, and finally obtained nanocarriers, i.e., RRMSN/DOX that can transport adriamycin (DOX) with high efficiency. Such nanodrug delivery system has a wide range of application prospects [11].

Zhang Yan et al. modified a negatively charged pH sensitive material, polyacrylic acid (PAA), on the MSN surface (PAA-MSNs) by acid-base conjugation, so as to block the mesoporous and achieve the response to pH environment. Then, the phospholipid bilayer (LP-PAA-MSNs) was coated on the surface of the PAA-MSNs to obtain a kind of “cell-alike” structure. This method can reduce the toxicity and aggregation of silica and effectively reduce the instant release effect of drugs on nanoparticles [12].

### 3.3. Application of Quantum Dots in the Medical Field

Carbon quantum dots (CQD_S_) are a kind of carbon-based zero-dimensional material. Compared with the traditional semiconductor quantum dots materials with high cost and large environmental damage, CQD_S_ not only have the characteristics of low production cost but also have many other excellent properties, such as small size, low toxicity, excellent water solubility, and environmental friendliness [13, 14] Therefore, CQDs has a wide application prospect and outstanding application value in biomedicine and biological imaging fields.

Cai et al. prepared a novel nanometer photosensitizer cerium dope carbon quantum dots (Ce-doped CDS) with high photothermal conversion efficiency. Ce-doped CD_S_ has good photothermal conversion performance. Because tumor cells are sensitive to temperature change, the photothermal killing effect is more significant. In cell viability experiments on MEF cells (mouse embryonic fibroblast cell line) and 4TI cells (human breast cancer cell line) irradiated with the same near-infrared laser (808 nm, 1.0 W/cm^2^) at 1200 *μ*g/mL of Ce-doped CD_S_, a 79% survival rate of MEF cells was observed, while only 7% of 4TI cells were left. The experiment also proved that the photothermal conversion efficiency of Ce-doped CDs is positively correlated with the concentration of Ce-doped CDs and the power density of infrared laser. The photothermal conversion ability of CDs is outstanding, and it can play a role only at lower concentration or with power density [15].

In terms of drug delivery, Wang et al. passivated the surfaces of graphene quantum dots (GQDs) with polyethylene glycol (PEG), obtained PEG-functionalized CQD_S_-PEG, and then loaded DOX onto the surface of CQD-PEG by hydrogen bond. The drug carrier DOX-GQDs-PEG was obtained. Its carrier capacity was significantly enhanced, and the release of the drug could be controlled by the change of pH. The killing effect of DOX-GQDS-PEG on cancer cells was significantly better than that of free DOX [16].

## 4. Application of Organic Nanomaterials in the Medical Field

### 4.1. Application of Nanoliposomes in the Medical Field

Liposome is a kind of self-assembled hollow balloon formed from phospholipid molecules. Due to its composition and structure similar to the cell membrane, it has excellent biocompatibility and it can play a good role in protecting and releasing the loaded drugs. Its particle size that is between 20 nm and 200 nm can be regulated [17, 18]. Smaller size is easy to be metabolized and degraded by human lytic enzymes, so that drugs can be well absorbed by the human body. In addition, the liposomes used as drug carriers can target drug delivery, improving efficacy and reducing toxic and side effects, which has a broad prospect in the development of anticancer nanodrugs [19, 20].

C_18_H_17_NO_6_ is a kind of anticancer substance extracted from sunglo. But its water solubility is poor, which has a certain influence on the efficacy. Fan et al. prepared nanoliposomes by combining C_18_H_17_NO_6_ with soybean phospholipids and cholesterol. The experiments on tumor transplantation in nude mice showed that the relative tumor proliferation rate (RPR) of nude mice decreased with the increase in the times of drug administration. And, there was a significant positive correlation between them. When the concentration of drug-loaded nanoliposomes was 4 *μ*g/ml, the maximum inhibitory rate of cell proliferation was less than 99.95%, which provided a direction for the research and development of new drugs. [21].

Papahadjopoulous et al. discovered and named cochleates [22] in their study of cationic-induced phospholipid membrane fusion in 1975. Cochleates are supramolecular autopolymers based on lipids and are long tubular structures formed by the curl of a negatively charged phospholipid bilayer, mediated by positive electric bridge agents. Due to the compact structure of the lipid coil, there is almost no aqueous phase inside it. This provides high stability during drug delivery and also prevents drug oxidation. In addition, cochleates can also achieve targeted delivery of cancer drugs. Since their characteristics are similar to cell membranes, they are easy to be engulfed by macrophages. However, since they are easy to aggregate and of large particle size and high production cost, a lot of work still needs to be done.

### 4.2. Application of Graphene in the Medical Field

Graphene oxide is generated from graphene nanomaterials under oxidation conditions. The surface and edge of the material are rich in oxygen-containing groups such as carbonyl group, hydroxyl group, and epoxy group. Due to these characteristics, functional graphene oxide nanomaterial can be constructed by modifying other active groups on the surface. At the same time, graphene oxide material has good water solubility and good biocompatibility. It has been widely used as a drug carrier in the biomedical field.

In 2008, Dai's research group successfully loaded insoluble aromatic structure antitumor drugs using graphene oxide. Experiments showed that graphene oxide could improve the solubility of insoluble loaded drugs, while the anticancer drugs in the complex still remained highly active, effectively killing cancer cells [23]. In addition, after modifying PEG on the surface of functional graphene oxide, the cytotoxicity of the carrier was reduced significantly and the biosafety was strong [24].

On this basis, Chen et al. found that graphene oxide has a high specific surface area, making its drug loading rate reach 238%, higher than that of ordinary nanomaterial carriers. In particular, it shows a super-high loading performance for DOX, the drug loading rate of which can reach 400%. There were also differences in drug release kinetics of functional graphene oxide under different pH conditions, which provides a theoretical basis for controlled release of the drug.

### 4.3. Application of Metal-Organic Frame Materials in the Medical Field

Metal-organic frameworks (MOFs) are a kind of hybrid materials assembled by organic ligands with metal ions or metal clusters. They have the advantages of large specific surface area, porous and adjustable pore size, good biocompatibility, adjustable composition, modifiable surface, etc., which provides a very great prospect for development of the carrier for drug delivery in the field of cancer therapy [25, 26].

The specific open metal sites and organic functional groups in MOFs can enhance the interaction between MOFs and drug molecules, so as to achieve controlled release of drugs and improve the delivery efficiency. Horcajada et al. used Cr-based MIL (Materials of Institute Lavoisier)-53/100/101 material to study the properties of loading and releasing of ibuprofen. In the simulated human environment, due to the difference of pore structure and drug action, the release time of MIL-53/100/101 is 20 days, 3 days, and 6 days, respectively. The drug load of MIL-101 is four times of MIL-100's. It is worth noting that MIL-53 is a flexible mesoporous material, whose drug release is up to 100%. [27].

There are also studies showing that zeolitic imidazolate frameworks (ZIF_S_) also have a great potential for development in the small molecule drug delivery. Among these, ZIF-8 is a metal framework composed of Zn and 2-methylimidazole, with a large and regulated aperture [28]. Zheng et al. synthesized a Zn-DOX-ZIF-8 nanoparticle, which is with good biocompatibility, good dispersion and stability, and good pH responsiveness, and whose curative effect for breast cancer is better than DOX drug alone [29].

## 5. Conclusion and Prospect

At present, nanomaterial, as a new type of material with a large specific surface area and good biocompatibility and degradability, has great potential for development in medical fields such as drug carriers, disease treatment, and artificial organs. Since the characteristics of different nanomaterials are quite different, this paper concluded and summarized the nanomaterials that are widely used at present (Table 1). At present, nanomaterials are widely used as drug carriers. Compared to traditional drug carrier, nanodrug carriers can be targeted to deliver drugs, improving the solubility and absorption rate of indissolvable drugs and reducing drug dosage. At the same time, nanomaterials can also generate synergistic effect with drugs, improving efficacy and reducing adverse reactions of the original medicine.

There are many materials still in the research stage and cannot be used in clinical practice. Although several widely used materials are mentioned above, some difficulties have not been solved, such as high production cost and certain cytotoxicity of materials. Nanomaterials, however, still showed potential in the medical field compared to the huge advantage of traditional materials. They have a bright application prospect in the fields of cardiovascular disease, antiviral therapy, rheumatoid arthritis, and obesity, in addition to the wide application in the field of antitumor. It is believed that, through the tireless efforts of researchers around the world, more new nanomaterials with good functions and high utilization will be applied in real life, with more profound development of nanomaterials and exploration. This article provides readers with cutting-edge knowledge about nanomedicine and a new understanding of nanomaterials.

## Figures and Tables

**Table 1:** Classification and property of nanomaterials.

Classification	Name	Property
Inorganic nanomaterials	Selenium nanoparticles	With good water solubility and fast degradation
	Mesoporous silica	With adjustable aperture, which is easy for synthesis and modification
	Quantum dots	At low production cost, of small size, with photothermal response

Organic nanomaterials	Liposomes	With controlled particle size, membrane-alike structure, and low cytotoxicity
	Graphene oxide	It has a large number of modification sites, diverse functions and strong extensibility, good dispersion, strong stability, and it can extend the cycle time
	Metal-organic frame	

## Data Availability

The datasets used and/or analyzed during the current study are available from the author on reasonable request.

## References

[1] FDA USA (2014). *Administration, Considering whether an FDA-Regulated Product Involves the Application of Nanotechnology FDA*.

[2] Kumar R., Aadil K. R., Ranjan S., Kumar V. B. (2020). Advances in nanotechnology and nanomaterials based strategies for neural tissue engineering. *Journal of Drug Delivery Science and Technology*.

[3] Qiu M., Singh A., Wang D. (2019). Biocompatible and biodegradable inorganic nanostructures for nanomedicine: silicon and black phosphorus. *Nano Today*.

[4] Ren H., Wan F., Dou K. (2018). Nano-drug. *Knowledge of Modern Physics*.

[5] Liu Q., Das M., Liu Y., Huang L. (2018). Targeted drug delivery to melanoma. *Advanced Drug Delivery Reviews*.

[6] Tong C. (2008). *Methods Study on Preparation and Characterization of Novel Biomaterials Based on Selenium Nanoparticles*.

[7] Gao X., Zhang J., Zhang L. (2000). Acute toxicity and bioavailability of nanometer red element selenium. *Health Research*.

[8] Ren S., Zhao X., Lin Y. (2020). The effect of selenium nanoparticles on the treatment of rheumatoid arthritis in rats. *Journal of Prenatal Diagnosis and Therapy*.

[9] Teng I.-T., Chang Y.-J., Wang L.-S. (2013). Phospholipid-functionalized mesoporous silica nanocarriers for selective photodynamic therapy of cancer. *Biomaterials*.

[10] Guo D. (2019). *Construction of Nano-Drug Delivery System Based on Mesoporous Silica and the Application in Tumor Combination Therapy*.

[11] Xiao D., Jia H.-Z., Ma N., Zhuo R.-X., Zhang X.-Z. (2015). A redox-responsive mesoporous silica nanoparticle capped with amphiphilic peptides by self-assembly for cancer targeting drug delivery. *Nanoscale*.

[12] Zhang Y., Fei W., Tao J., Zou J., Lu Y., Li F. (2018). Angiopep-2 modified mesoporous silica lipocyst nanoporous drug delivery system with arsenic trioxide and in vitro. *Chinese Traditional and Herbal Drugs*.

[13] Shen J., Zhu Y., Yang X., Li C. (2012). Graphene quantum dots: Emergent nanolights for bioimaging, sensors, catalysis and photovoltaic devices. *Chemical Communications*.

[14] Fang Y., Guo S., Li D. (2012). Easy synthesis and imaging applications of cross-linked green fluorescent hollow carbon nanoparticles. *ACS Nano*.

[15] Cai R., Zhang J., Xu L. (2020). Construction of novel cerium doped carbon quantum dots and its killing effect on breast cancer cells. *Journal of Jiangsu University*.

[16] Wang K. (2017). *Preparation of Graphene Quantum Dots and Their Applications in Biological Imaging and Drug Delivery*.

[17] Haeri A., Osouli M., Bayat F., Alavi S., Dadashzadeh S. (2018). Nanomedicine approaches for sirolimus delivery: a review of pharmaceutical properties and preclinical studies. *Artificial Cells, Nanomedicine, and Biotechnology*.

[18] Liu Q., Zhang J., Sun W., Xie Q. R., Xia W., Gu H. (2012). Delivering hydrophilic and hydrophobic chemotherapeutics simultaneously by magnetic mesoporous silica nanoparticles to inhibit cancer cells. *International Journal of Nanomedicine*.

[19] Tardi P., Choice E., Masin D., Redelmeier T., Bally M., Madden T. D. (2000). Liposomal encapsulation of topotecan enhances anticancer efficacy in murine and human xenograft models. *Cancer Research*.

[20] Torchilin V. P. (2005). Recent advances with liposomes as pharmaceutical carriers. *Nature Reviews Drug Discovery*.

[21] Fan D., He X., Li R. (2019). Study on the antitumor effect of natural active compound C18H17NO6 nanoliposomes. *Chinese Traditional and Herbal Drugs*.

[22] Papahadjopoulos D., Vail W. J., Jacobson K., Poste G. (1975). Cochleate lipid cylinders: formation by fusion of unilamellar lipid vesicles. *Biochimica et Biophysica Acta (BBA) - Biomembranes*.

[23] Liu Z., Robinson J. T., Sun X., Dai H. (2008). PEGylated nanographene oxide for delivery of water-insoluble cancer drugs. *Journal of the American Chemical Society*.

[24] Yang X., Zhang X., Liu Z., Ma Y., Huang Y., Chen Y. (2008). High-efficiency loading and controlled release of doxorubicin hydrochloride on graphene oxide. *Journal of Physical Chemistry C*.

[25] Horcajada P., Gref R., Baati T. (2012). Metal-organic frameworks in biomedicine. *Chemical Reviews*.

[26] Keskin S., Kizilel S. (2011). Biomedical applications of metal-organic frameworks. *Industrial & Engineering Chemistry Research*.

[27] Horcajada P., Serre C., Vallet-Regí M., Sebban M., Taulelle F., Férey G. (2006). Metal-organic frameworks as efficient materials for drug delivery. *Angewandte Chemie International Edition*.

[28] Zheng H., Zhang Y., Liu L. (2016). One-pot synthesis of metal-organic frameworks with encapsulated target molecules and their applications for controlled drug delivery. *Journal of the American Chemical Society*.

